# Evaluating the Implementation Fidelity of a Motivational Health Coaching Intervention to Improve Self-Care for Caregivers of Persons Living With Dementia

**DOI:** 10.1177/07334648251322548

**Published:** 2025-02-26

**Authors:** Lauren Fisher, Lauren Massimo, Barbara Riegel, Tracie J. Walser, Karen B. Hirschman

**Affiliations:** 1 6572 University of Pennsylvania, Philadelphia, PA, USA

**Keywords:** caregiving, dementia, intervention, support, self-care, implementation science

## Abstract

Frontotemporal degeneration (FTD) is one of the leading causes of early-onset dementia, causing a progressive deterioration in patient cognition and function. These changes often lead to increased caregiver burden and health self-care neglect due to increased focus on the needs of the person living with FTD. This study aimed to evaluate implementation of an evidence-based virtual health coaching intervention designed to improve self-care of FTD caregivers. Guided by the Consolidated Framework for Intervention Fidelity, adherence to the intervention (exposure and content) was measured with a total score ranging from 6 (low adherence) to 18 (high adherence). Overall, about half the sessions were deemed high adherence, with a gradual decrease in total adherence over time, primarily due to decreases in exposure adherence. Our results reflect the anticipated variation in sessions to maintain person-centered care. Overall, a virtual health coaching intervention for FTD caregivers can be delivered with relatively high adherence.

**What this paper adds**

• There are many evidence-based interventions available for caregivers of persons living with dementia; yet, more research on the evaluation of implementation fidelity of evidence-based interventions is needed
• Preliminary evidence that a virtual health coaching intervention for caregivers of persons living with FTD can be delivered with relatively high adherence.

**Applications of study findings**

• This study demonstrates a feasible way of measuring adherence to a protocol that may be applied to other RCTs
• The results demonstrate that potential adaptations of the intervention are needed as caregivers become more adept at the intervention, leading to less total time.

## Background

Behavioral variant frontotemporal degeneration (bvFTD) is a form of early-onset dementia with hallmark symptoms of progressive changes in personality and behavior (Cosseddu et al., 2020). Due to the young age of onset and disabling symptoms, persons living with FTD are often unable to care for themselves independently and require significant assistance from a caregiver, most often a spouse or adult child (Massimo & Grossman, 2008), leading to high levels of distress, burden, and depression for FTD caregivers (Liu et al., 2017; Uflacker et al., 2016). A recent meta-analysis of caregiver interventions tailored for heterogeneous forms of dementia highlighted the value of multicomponent interventions (Walter & Pinquart, 2020). However, little to no information is provided on the implementation fidelity of these interventions. Implementation fidelity is the degree to which an intervention is implemented or enacted as intended (Carroll, 2020). Examining the implementation fidelity of evidence-based interventions (EBIs) is essential to understand what elements of the intervention may be influencing the outcomes (Carroll, 2020). The aim of this paper is to describe the implementation fidelity of an EBI designed to improve self-care in caregivers of people living with bvFTD (bvFTD caregivers) as part of a pilot randomized controlled trial (RCT).

## Methods

Implementation evaluation of the delivery of the intervention occurred concurrently with the RCT pilot study described below.

### Overview of the Parent Pilot Trial

The pilot study evaluated the preliminary efficacy of a self-care virtual intervention, Virtual Caregiver Coach for You (ViCCY), for bvFTD caregivers compared to an active control group that received virtual health information (HI) only. Findings from this pilot indicated significant improvement in self-care monitoring and self-care confidence on the Self-Care Inventory, suggesting that caregivers who received ViCCY improved their self-care over the duration of the intervention (Massimo et al., 2023).

#### Sample and Group Assignment

bvFTD caregivers (hereafter, caregivers) were recruited from a neurology specialty clinic at the University of Pennsylvania. The goal was to recruit and equally randomize 30 caregivers to the control or intervention groups. See Table 1 for inclusion/exclusion criteria. Each enrolled participant completed a baseline interview and was then provided with an Apple iPad (with cellular data) to access preloaded websites with health information related to caregiving, stress, and bvFTD. iPads also linked to a secure video conferencing room for health coaching sessions.

**Table 1.** Inclusion and Exclusion Criteria for Pilot Study.

Inclusion criteria	Exclusion criteria
Provide at least 8 hours/week of informal caregiving to a person diagnosed with bvFTD	Untreated major psychiatric illness
Health self-care neglect scale score ≥2	Cognitive impairment
English speaking	Participation in another caregiving support clinical trial
Subjective comfortability using technology	

#### ViCCY Intervention

The ViCCY intervention consists of 10 virtual health coaching sessions over six months. Session topics included assessing caregiving demands and needs (Sessions 1 and 2), stressors and coping (Sessions 2 and 3), self-care (Sessions 3–7), and building support and resources (Sessions 8–10) (Riegel et al., 2019). While the health coaches followed a specific manual, they could individualize the content as needed for individual caregivers. Each session was designed to last approximately 60 minutes. Results presented here pertain to implementation fidelity for the intervention arm only.

### Framework

**Figure 1.**
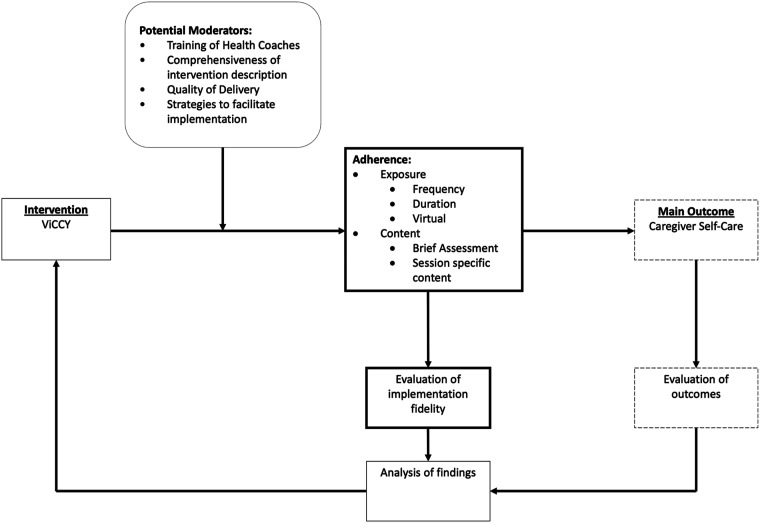
Adapted Conceptual Framework for Implementation Fidelity of Virtual Caregiver Coach for You (ViCCY). Notes: Adapted conceptual framework for implementation fidelity (Carroll, 2020) for Virtual Caregiver Coach for You (ViCCY). This model demonstrates the framework for evaluating adherence to the intervention, including exposure and content. This paper reports findings related to adherence to exposure and content and the evaluation of implementation fidelity (bold boxes). Results corresponding to the dashed line boxes are reported elsewhere (Massimo et al., 2023).

The Consolidated Framework for Fidelity (CFIF) (Carroll, 2020) and adherence measurement techniques described by Bunker et al. (2024) were adapted for this study. Using CFIF, adherence was assessed as: (1) Exposure—frequency of contacts, length of contacts, and use of technology; and (2) Content of the intervention—what information was delivered. For this pilot, coverage is not presented since the recruitment goal (*N* = 30, 15 per group) was previously reported (Massimo et al., 2023). How these specific elements are related to this study is shown in Figure 1.

### Data Collection

Each ViCCY session was audio-recorded with participant consent and uploaded to a secure, password-protected cloud-based server. Health coaches tracked their time in minutes for all participant contacts (e.g., outreach, sessions) in a time log. After each session, health coaches documented session-specific comments capturing the topics discussed, their overall perception of the session, and technology use (e.g., video and audio quality rating of good, fair, or poor).

#### Measures

For the subcategories of adherence—exposure (frequency [count], duration [minutes], and technology [use and quality]) and content—guiding questions and specific units of measure were used to determine the level of adherence (see Table 2).

**Table 2.** Dimensions of Adherence and Measuring Implementation Fidelity to the Intervention.

Adherence subcategory	Intervention component	Guiding question	Measure	Data sources	Scores assigned
Exposure					
Frequency	Individual session	How frequently did participants receive each individual session?	By session, how many participants received the session on its own and not merged with other topics Expected: Each participant would have at least 9 separate sessions	Health coach time log	Separate sessions 90%–100%: 3 75%–89%: 2 <75: 1
Duration	How long	How long was each health coaching session?	By session, the median duration of contact (minutes) to complete specific session content Expected: No less than 54 minutes	Health coach time log	>/= 54 minutes: 3 45–53 minutes: 2 <45 minutes: 1
Technology	What device	By what technological device was the intervention delivered?	By session the type of the technology used for each session (e.g., iPad Zoom, cell phone call, cell phone FaceTime) Expected: 90% iPad Zoom use	Session-specific document Audio recordings	90%–100% iPad: 3 75%–89% iPad: 2 <75% iPad: 1
	Device video and audio quality	How was the audio and video quality?	By session, the overall audio and video quality (good vs. fair/poor) Expected: 90% good audio/video	Session-specific document Audio recordings	90%–100% scored good : 3 75%–89% : 2 <75% : 1
Content	Brief assessment	Did participants receive the expected brief assessment at each session?	By session, did health coaches address all pertinent brief assessment questions Expected: At least 90% fidelity	Audio recordings	90%–100%: 3 75%–89%: 2 <75: 1
	Session-specific	Did participants receive the expected session-specific content for each session?	By session, did health coaches address all pertinent session-specific content and questions Expected: At least 90% fidelity	Audio recordings	90%–100%: 3 75%–89%: 2 <75: 1

Notes: Table 2 displays each adherence subcategory and specifically how the domain was measured. To unify the scoring, a scoring schema of low (1: less than 75% adherence), medium (2: 75–89% adherence), and high (3: greater than 90% adherence) was utilized to depict whether sessions met idealized fidelity. To calculate the scoring thresholds for duration, the percent thresholds were converted into minutes utilizing 60 minutes as the expected (100%) adherence value.

Content is defined by the information presented to participants in each health coaching session. The content was broken into (1) Brief Assessment (e.g., Did the health coach ask about the caregiver’s mood using a numeric scale?) and (2) Session-Specific Content (e.g., Did the health coach ask how aware the caregiver is that they are experiencing stress?). To evaluate content adherence, audio recordings were reviewed by two raters (LF and KBH) who listened to 28% (40/142) of the session recordings and independently assessed whether the content was delivered as intended using a standardized form. Coded sessions were reviewed and consensus agreement was used to finalize the fidelity scores. The content fidelity scores were calculated based on the sum of the possible elements (0-not present, 1-partially present, 2-present) observed in a specific session and divided by the total possible elements score for that session (e.g., if there were eight possible elements, the highest score was 16; 8 elements X 2 [present] equals a score of 16) and then the score was divided by the possible total score (e.g., 16/16 = (1) and multiplied by 100 to create a standardized percentage of fidelity met for each session (e.g., 100%). The content fidelity scores were calculated per session and then summed across all participants for a total adherence score across all completed sessions and health coaches.

To unify the scoring across adherence subcategories, a scoring schema of low (1: less than 75% adherence), medium (2: 75%–89% adherence), and high (3: greater than 90% adherence) was utilized to depict whether sessions met idealized fidelity (adapted from Bunker et al., 2024). For duration (captured in minutes), adherence was calculated by taking the corresponding percent stated above for the idealized time of 60 minutes. For example, a high adherence score (greater than 90%) was calculated by taking 90% of the idealized session time of 60 minutes, resulting in 54 minutes. Therefore, the scoring scheme for duration is low (1: <45 minutes), medium (2: 45–53 minutes), and high (3: >54 minutes).

### Data Analysis

Basic descriptive statistics (e.g., counts/percentages, means/standard deviations) were calculated and presented.

## Results

Table 3 presents each adherence subcategory as raw data in their unique units of measure. Taking the data from Table 3, we applied the level of adherence framework adapted from Bunker et al. (2024) described in the Measures section to create unified scores and present those findings in Table 4.

**Table 3.** Adherence to Implementing the ViCCY Intervention With Fidelity (Raw Data).

Adherence subcategory	Intervention component	Unit of measure	Session									
			1	2	3	4	5	6	7	8	9	10
Frequency	Individual sessions	*N* participants who completed each individual session / total participants who completed the session content	15/15	15/15	14/15	13/15	9/14	9/14	8/14	7/14	5/13	5/13
Duration	How long	Median minutes per session	100	65	60	60	49.5	46.5	32.5	37.5	30	20
Technology	What device	*N* participants who completed session on iPad tablet/ total participants who completed the session	12/15	13/15	14/15	14/15	13/14	13/14	13/14	12/14	12/13	11/13
	Quality	% Scored as “good” audio and video quality	90	97	97	100	96	96	93	96	85	96
Content	Brief assessment	% Adherence to completing a brief assessment	92	96	97	100	99	100	100	100	98	96
	Session-specific	% Adherence to specific session content	94	100	92	100	85	100	85	100	94	90

Notes: This table displays the raw data scores with units of measurement for each adherence category. Frequency: the denominator includes all participants who completed a specific session, and the numerator includes only those who completed the content as a unique individual session.

**Table 4.** Standardized Assessment of Level of Adherence to Key Intervention Components, by Session.

Adherence subcategory	Intervention component	Session									
		1	2	3	4	5	6	7	8	9	10
Frequency	Individual sessions	3	3	3	2	1	1	1	1	1	1
Duration	How long	3	3	3	3	2	2	1	1	1	1
Technology	What device	2	2	3	3	3	3	3	2	3	2
	Quality	3	3	3	3	3	3	3	3	2	3
Content	Brief assessment	3	3	3	3	3	3	3	3	3	3
	Session-specific	3	3	3	3	2	3	2	3	3	3
Totals (max. 18)		17	17	18	17	14	15	13	13	14	13

Notes: This table displays the converted raw data scores from Table 3 into the standardized adherence scores (each component range 1–3; total score range 6–18).

### Exposure

#### Frequency

Sessions 1–3 maintained high adherence, while Session 4 fell to medium adherence. The combination of sessions resulted in low adherence for sessions 5–10, with less than 75% of the participants having those sessions as independent meetings. The sessions combined most often were sessions 5 with 6 (both related to self-monitoring) and 9 with 10 (both pertaining to building support and resources).

#### Duration

On average, sessions were 52 minutes long (+/−32 minutes), with early sessions being longer than later sessions (see Table 3). High adherence (>/= 54 minutes) was achieved for sessions 1–4, medium adherence (45–53 minutes) for sessions 5–6, and low adherence (<45 minutes) for sessions 7–10.

#### Technology

Overall, the technology quality maintained high adherence for all sessions. A few sessions (1, 2, 8, and 10) were conducted using alternative technology methods (e.g., Facetime) resulting in medium adherence.

### Content

All sessions maintained high adherence (>90% fidelity) for brief assessment, see Table 4. Similarly, high adherence was achieved for session-specific content for all sessions, except Sessions 5 (self-monitoring) and 7 (relaxation), which maintained medium adherence (75%–89% fidelity).

## Discussion

Overall, this pilot study showed mixed adherence across the sessions, with most sessions demonstrating high adherence. These fidelity results reinforce the outcomes of the pilot trial, which indicated improvements in self-care outcomes for the intervention group compared to the control group (Massimo et al., 2023). Based on this evaluation, we are confident that the intervention was delivered as intended, further supporting the significant positive outcomes observed in the intervention group.

First, over half of the participants had at least one session merged with another, resulting in low fidelity for the frequency of individualized session contacts. While the protocol intended to complete each session as a stand-alone topic, due to time constraints, participant preference, and health coach assessment, health coaches combined at least two sessions for 9 out of 15 participants. Sessions 5 and 6 (coping and self-monitoring) and sessions 9 and 10 (addressing unmet needs and reinforcement) were most frequently combined. It is important to highlight that the content from each merged session was evaluated independently for adherence. High fidelity was maintained concerning the content even when sessions were merged. Future implementation of ViCCY should consider allowing for merging of some sessions as needed.

Second, session duration decreased over time. To best receive the intervention, it is possible that some caregivers needed more time to debrief and form a connection in the beginning (Sessions 1 and 2). Over time, their established relationship resulted in briefer sessions as less time was spent on caregiver background. Likely, the fact that some sessions were merged may also have decreased session time. For example, when sessions 9 and 10 were merged, though the total time of the conversation remained around 50 minutes, health coaches reported spending approximately 30 minutes on session 9 and 20 minutes on session 10, resulting in low adherence for those individual sessions.

Third, the technology in this study (Apple iPads) proved stable in maintaining connectivity and limiting technical problems secondary to unstable operating systems that challenged a previous trial of ViCCY (Hirschman et al., 2020). In the few instances when the iPad was not used for the coaching session, it was secondary to caregiver preference (i.e., FaceTime or telephone). Flexibility in delivery should be considered for future studies delivering virtual interventions to allow participants to use their own device.

Overall, there was strong adherence to delivery of the protocol content, as evidenced by high adherence to the brief assessment and session-specific content. Though the brief assessment adherence remained high for all sessions, session-specific content adherence did fall to medium adherence in some sessions (specifically Sessions 5 and 7). Session-specific content was designed to allow for flexibility in intervention delivery, allowing the health coaches to tailor the intervention for the participants based on caregiver needs. Overall, the high adherence to content may be related to the health coaches who were already familiar with the protocol through their work in coaching heart failure caregivers and the detailed session-by-session manual provided (Hirschman et al., 2020).

### Limitations

This pilot study was designed to test the preliminary efficacy of the intervention with a small sample of bvFTD caregivers, which limits study generalizability. However, the techniques described here to assess fidelity to the intervention can be replicated with larger samples to assess the quality and consistency of implementation. While trained study staff (LF, KBH) completed fidelity assessments continuously, their own biases could have been introduced during scoring. To minimize bias, the team completed thorough training and met frequently to review fidelity scoring and reach a consensus on any coding discrepancies.

### Implications for Future Trials

This study demonstrates the importance of providing detailed information related to implementation fidelity to ensure trustworthy replication and a clear demonstration of the mechanism of action by the intervention. Considerations for future studies include evaluating the effect of adherence score on primary outcomes, the importance of individualized (as opposed to merged) sessions, and implications in a larger sample.

### Conclusion

Though the frequency and duration of the health coaching sessions varied, many of the other key aspects maintained high adherence. It is evident that as the intervention progressed over time, modifications may have been made to maintain person-centeredness, thus minimizing overall adherence to the protocol.

## References

[bibr3-07334648251322548] Bunker J. N. Hilgeman M. M. McCreedy E. Gadbois E. Thomas K. S. (2024). Evaluating the implementation fidelity of a pilot pragmatic randomized clinical trial comparing daily-delivered meals to mailed frozen meals. Journal of Applied Gerontology, 43(11), 1605–1610. 10.1177/07334648241248269 38686741

[bibr4-07334648251322548] Carroll C. (2020). Fidelity. In Handbook on implementation science (pp. 291–316). Edward Elgar Publishing Limited.

[bibr5-07334648251322548] Cosseddu M. Benussi A. Gazzina S. Alberici A. Dell’Era V. Manes M. Cristillo V. Borroni B. Padovani A. (2020). Progression of behavioural disturbances in frontotemporal dementia: A longitudinal observational study. European Journal of Neurology, 27(2), 265–272. 10.1111/ene.14071 31448481

[bibr6-07334648251322548] Hirschman K. B. Bowles K. H. Garcia-Gonzalez L. Shepard B. Walser T. J. Thomas G. L. Stawnychy M. A. Riegel B. (2020). Lessons learned from the implementation of a video health coaching technology intervention to improve self-care of family caregivers of adults with heart failure. Research in Nursing & Health, 44(1), 250–259. 10.1002/nur.22100 33341950 PMC8486377

[bibr7-07334648251322548] Liu S. Jin Y. Shi Z. Huo Y. R. Guan Y. Liu M. Liu S. Ji Y. (2017). The effects of behavioral and psychological symptoms on caregiver burden in frontotemporal dementia, Lewy body dementia, and Alzheimer’s disease: Clinical experience in China. Aging and Mental Health, 21(6), 651–657. 10.1080/13607863.2016.1146871 26882509

[bibr8-07334648251322548] Massimo L. Grossman M. (2008). Patient care and management of frontotemporal lobar degeneration. American Journal of Alzheimer’s Disease and Other Dementias, 23(2), 125–131. 10.1177/1533317507307961 PMC2927812 18421010

[bibr9-07334648251322548] Massimo L. Hirschman K. B. Aryal S. Quinn R. Fisher L. Sharkey M. Thomas G. Bowles K. H. Riegel B. (2023). iCare4Me for FTD: A pilot randomized study to improve self-care in caregivers of persons with frontotemporal degeneration. Alzheimer’s and Dementia: Translational Research & Clinical Interventions, 9(2), Article e12381. 10.1002/trc2.12381 PMC10152138 37143583

[bibr10-07334648251322548] Riegel B. Hanlon A. L. Coe N. B. Hirschman K. B. Thomas G. Stawnychy M. Wald J. W. Bowles K. H. (2019). Health coaching to improve self-care of informal caregivers of adults with chronic heart failure – iCare4Me: Study protocol for a randomized controlled trial. Contemporary Clinical Trials, 85(September), Article 105845. 10.1016/j.cct.2019.105845 31499227 PMC6815729

[bibr11-07334648251322548] Uflacker A. Edmondson M. C. Onyike C. U. Appleby B. S. (2016). Caregiver burden in atypical dementias: Comparing frontotemporal dementia, Creutzfeldt-Jakob disease, and Alzheimer’s disease. International Psychogeriatrics, 28(2), 269–273. 10.1017/S1041610215001647 26435062

[bibr12-07334648251322548] Walter E. Pinquart M. (2020). How effective are dementia caregiver interventions? An updated comprehensive meta-analysis. The Gerontologist, 60(8), 609–619. 10.1093/geront/gnz118 33226434

